# Durability Assessment of a Plasma-Polymerized Coating with Anti-Biofilm Activity against *L. monocytogenes* Subjected to Repeated Sanitization

**DOI:** 10.3390/foods10112849

**Published:** 2021-11-18

**Authors:** Ignacio Muro-Fraguas, Paula Fernández-Gómez, Rodolfo Múgica-Vidal, Ana Sainz-García, Elisa Sainz-García, Márcia Oliveira, Montserrat González-Raurich, María López, Beatriz Rojo-Bezares, Mercedes López, Fernando Alba-Elías

**Affiliations:** 1 Department of Mechanical Engineering, University of La Rioja, C/San José de Calasanz 31, 26004 Logroño, Spain; ignacio.muro@unirioja.es (I.M.-F.); ana.sainz@unirioja.es (A.S.-G.); elisa.sainzg@unirioja.es (E.S.-G.); fernando.alba@unirioja.es (F.A.-E.); 2 Department of Food Hygiene and Technology, Institute of Food Science and Technology, Campus de Vegazana s/n, Universidad de León, 24071 León, Spain; pafeg@unileon.es (P.F.-G.); msouo@unileon.es (M.O.); mmgonr@unileon.es (M.G.-R.); mmlopf@unileon.es (M.L.); 3 Molecular Microbiology Area, Center for Biomedical Research of La Rioja (CIBIR), C/Piqueras 98, 26006 Logroño, Spain; mlopezm@riojasalud.es (M.L.); brojo@riojasalud.es (B.R.-B.)

**Keywords:** durability, anti-biofilm, plasma polymerization, sanitization, disinfectants, food contact

## Abstract

Biofilm formation on food-contact surfaces is a matter of major concern causing food safety and spoilage issues to this sector. The aim of this study was to assess the durability of the anti-biofilm capacity of a plasma-polymerized coating composed of a base coating of (3-aminopropyl)triethoxysilane (APTES) and a functional coating of acrylic acid (AcAc). Coated and uncoated AISI 316 stainless steel (SS) plates were subjected to five sanitization cycles with sodium hypochlorite (0.05%) and peracetic acid (0.5%). The effectiveness of the coating for the inhibition of multi-strain *Listeria monocytogenes* biofilm formation was confirmed using a three-strain cocktail, which was grown on the SS plates at 12 °C for 6 days. Compared to the uncoated SS, relative biofilm productions of 14.6% on the non-sanitized coating, 27.9% on the coating after sanitization with sodium hypochlorite, and 82.3% on the coating after sanitization with peracetic acid were obtained. Morphological and physicochemical characterization of the coatings suggested that the greater anti-biofilm effectiveness after sanitization with sodium hypochlorite was due to the high pH of this solution, which caused a deprotonation of the carboxylic acid groups of the functional coating. This fact conferred it a strong hydrophilicity and negatively charged its surface, which was favorable for preventing bacterial attachment and biofilm formation.

## 1. Introduction

In order to guarantee food safety, inactivating pathogenic microorganisms and controlling their occurrence in food processing environments is of utmost importance in the food industry. Among the most concerning foodborne pathogens, *Listeria monocytogenes* stands out as one of the most difficult to control and eliminate due to its ubiquity and the capacity to grow in anaerobic conditions and at refrigeration temperatures. Furthermore, *L. monocytogenes*, which has been implicated in numerous foodborne disease outbreaks, is capable of forming biofilms, which are communities of bacteria attached to a surface and embedded in a matrix of self-produced extracellular polymeric substances (EPS) [1]. Biofilms are an important problem in food industries because they enable bacteria to adhere to a wide variety of materials that are frequently used in food-contact applications such as plastics, stainless steel and glass, and they provide bacteria with protection against antimicrobial agents and stressful environments [2,3]. As a result, bacteria in biofilms are more prone to develop tolerance to disinfectants than planktonic bacteria and are involved in persistence events. This implies a greater risk of cross-contamination of food, leading to food spoilage and posing a threat to public health [4].

A common practice for controlling biofilm formation in the food industry is to periodically sanitize the surfaces with chemical agents with antimicrobial activity (e.g., sodium hypochlorite (NaOCl), peracetic acid (C_2_H_4_O_3_), sodium hydroxide (NaOH), or hydrogen peroxide (H_2_O_2_)) [5]. More specifically, sodium hypochlorite is the most commonly used sanitizer due to its broad-spectrum action and the fact that it is easy to handle, cheap, widely available, and removes organic foulants [6,7,8]. Sodium hypochlorite produces hypochlorous acid (HOCl) and hypochlorite ion (-OCl), which can penetrate the cell membrane and kill bacteria by oxidizing their essential enzymes [3,9]. However, it produces chlorinated by-products that are potentially dangerous for human health. As a result, efforts have been made to reduce the use this sanitizer or replace it with other antibacterial approaches [10,11]. In this regard, peracetic acid is a widely used alternative in the food industry because it has significant bactericidal effects and it does not generate chlorinated by-products [12,13].

Nevertheless, due to the complexity of bacterial biofilms, and the associated enhanced ability to develop tolerance to sanitizers, it is difficult to achieve a complete elimination of biofilms in industry settings. One of the initial steps in biofilm formation consists in the attachment of bacteria to a surface, which is influenced by its physicochemical properties, including functional groups, topography, wettability, and electrostatic charge [3,5]. Considering this, many efforts have been made for producing surfaces with physicochemical properties that prevent biofilm formation by hindering the initial attachment of bacteria, and therefore, reducing the possibility of resistance development [14,15,16,17,18]. In our previous work [17], anti-biofilm coatings that inhibited biofilm formation by *L. monocytogenes* were deposited by an atmospheric-pressure plasma jet (APPJ) system on AISI 316 stainless steel (SS) using different precursors and process parameters. The most promising coating found in that previous study was composed of a base coating of (3-aminopropyl)triethoxysilane (APTES) and a functional coating of acrylic acid (AcAc) (coating AP10 + AA6). It reduced biofilm formation by a single strain of *L. monocytogenes* to ≈10% compared to the biofilm formation observed on uncoated SS after incubation at 12 °C, which resembled the conditions prevailing in food industries. According to the results obtained, it was suggested that the reduction in the initial attachment of bacterial cells was due to (1) the strong hydrophilicity of the coating, which would have promoted the formation of a water barrier, and (2) the smooth surface of the coating, which reduced the occurrence of potential sheltering sites.

In real industrial environments, multiple bacterial strains coexist, and the coated surfaces would still require periodical sanitization to prevent long-term bacterial accumulation and cross-contamination of food products. Therefore, in the present work, the anti-biofilm effectiveness of the coating AP10 + AA6 against multi-strain *L. monocytogenes* biofilm formation was verified, and its durability after repeated cycles of sanitization with commonly used disinfectants, such as sodium hypochlorite and peracetic acid, was also assessed.

## 2. Materials and Methods

### 2.1. Coating Process

An APPJ system PlasmaSpot500^®^ (MPG, Luxembourg) was used for coating stainless steel AISI 316 (SS) plates (Ø 35 mm) via plasma polymerization. The APPJ has two cylindrical electrodes that are arranged coaxially, as well as an Al_2_O_3_ dielectric barrier between them. The internal electrode is grounded, and the external one is connected to a high-voltage generator that operates at a frequency of 68 kHz, which was set at a power of 360 W for the deposition of the coating AP10 + AA6. A flow of nitrogen (99.999%) at 80 slm was used for the plasma generation. The SS plates were treated following a scanning pattern with a pitch of 2 mm and keeping a gap of 10 mm between the top of the plates and the plasma gun. Before coating, the SS substrates were activated by exposing them to one pass of the plasma jet at 50 mm/s without adding any precursor. Then, the coating AP10 + AA6 was deposited onto the activated SS plates in two plasma-polymerization stages. Firstly, APTES (Sigma-Aldrich Chemie GmbH, Steinheim, Germany) was added to the plasma jet for the deposition of a base coating by performing 10 passes at 50 mm/s. Secondly, AcAc (Merck, Darmstadt, Germany) was used for the deposition of a functional coating by performing 6 passes at 100 mm/s over the previously deposited base coating. The precursor liquids were atomized and carried by a flow of nitrogen (99.999%) at 1.5 slm. The precursors circulated along the interior of the inner electrode and were finally added perpendicularly to the plasma jet through a slit at the end of the electrode.

### 2.2. Sanitization Process

Uncoated and coated plates were subjected to sanitization with either peracetic acid (Merck, Darmstadt, Germany) or sodium hypochlorite (VWR, Radnor, PA, USA) solutions at 0.5% and 0.05%, respectively. These concentrations of the sanitizing solutions were selected because they are in the range of concentrations of the commercial disinfectants that are used in food industries. The pH values of the sanitizing solutions were measured using a Sension+ MM150 multimeter with a 5048 electrode (HACH, Bilbao, Spain), thus obtaining a pH of 3.1 for the peracetic acid solution and of 10.4 for the sodium hypochlorite solution. A succession of 5 sanitization cycles was applied using each of the two solutions. Each cycle comprised the following steps. First, uncoated and coated surface plates were covered with 6 mL of the sanitizing solution during 15 min. Then, the solution was discarded, and the plates were left to dry at room temperature conditions.

### 2.3. Biofilm Formation Quantification

Uncoated SS (positive control) and coated plates were submitted to biofilm formation assays in parallel. These assays were performed on plates that had been subjected to 5 sanitization cycles as described above as well as on plates that had not been sanitized with any of the solutions. A three-strain cocktail of *L. monocytogenes* containing the CECT911 reference strain and two strains previously isolated from the processing environment of a meat industry (ULE1264 and ULE1265) [19] was used. The bacterial strains were stored frozen, and they were revitalized previously to the preparation of the stock cultures. The bacterial inocula, which were prepared by transferring one isolated colony of the stock cultures from BHI agar plates into 10 mL of fresh BHI broth (Merck, Darmstadt, Germany) and incubating at 37 °C for 24 h, were mixed and diluted in fresh BHI broth to obtain a bacterial suspension with a cell density of approximately 10^6^ CFU/mL. For each experimental set up (sanitized or non-sanitized), four uncoated and four coated plates were inoculated with 4 mL of the bacterial suspension. A negative control containing non-inoculated BHI broth was included in all experiments. The plates were incubated for 6 days at 12 °C, which is a temperature that usually prevails in industrial environments and corresponds to the maximum recommended temperature for facilities processing products of animal origin according to the European Parliament and the Council of the European Union [20]. After incubation, biofilm formation was assessed by crystal violet (CV) staining, following the same procedure as in our previous work [17]. Briefly, the supernatant was discarded, and the plates were washed three times with Ringer solution (Merck, Darmstadt, Germany). Then, the biofilm was stained, adding 4 mL of a 0.1% CV solution (Panreac, Barcelona, Spain) and incubating at room temperature for 15 min. In order to remove the excess of stain, plates were washed three times with Ringer solution, and the cell-bound CV was dissolved by adding 5 mL of ethanol (Dávila Villalobos, Cabezón de Pisuerga, Spain) at 95% to each plate. After 15 min, the optical density at 595 nm (OD_595_) was measured with a UV-3100PC spectrophotometer (VWR, Radnor, PA, USA). The relative biofilm production (%) for each of the three sanitization scenarios (i.e., non-sanitized, sanitized with peracetic acid (0.5%), and sanitized with sodium hypochlorite (0.05%)) was calculated as follows:

$$\mathrm{Relative}\;\mathrm{biofilm}\;\mathrm{production}=(\frac{{\mathrm{OD}}_{595}\;\mathrm{coating}}{{\mathrm{OD}}_{595}\;\mathrm{SS}})\;\times \;100$$

where:

OD_595_ coating = mean OD_595_ for inoculated coated plates − mean OD_595_ for non-inoculated coated plates.

OD_595_ SS = mean OD_595_ for inoculated uncoated SS plates − mean OD_595_ for non-inoculated uncoated SS plates.

Relative biofilm productions <100% indicate an anti-biofilm effect.

### 2.4. Statistical Analysis

Differences between biofilm formation (OD_595_) on coated and uncoated SS, and be-tween the relative biofilm formation (%) for the different sanitization scenarios were evaluated using analysis of variance (ANOVA). Data were previously normalized by logarithmic transformation, and differences were considered statistically significant at *p* < 0.05. In the case of the relative biofilm formation analysis, the ANOVA test was followed by a multiple comparison test using Tukey HSD (*p* < 0.05). Statistical analyses were performed with R Studio (v 4.0.4).

### 2.5. Characterization of Samples

#### 2.5.1. Atomic Force Microscopy (AFM) Analysis

A quantitative morphological analysis of the uncoated SS and coated plates before and after 5 sanitization cycles with peracetic acid and sodium hypochlorite was done by AFM. Three areas of 40 µm × 40 µm of each sample were recorded by a Multimode atomic force microscope with a Nanoscope V Controller (Bruker Corporation, Billerica, MA, USA) working in tapping mode at a frequency of 50 Hz. The average roughness values (Ra) of the recorded areas were obtained using the software NanoScope Analysis 1.4 (Bruker Corporation, Billerica, MA, USA).

#### 2.5.2. Scanning Electron Microscopy (SEM) Analysis

A qualitative morphological analysis of the uncoated SS and coated plates before and after 5 sanitization cycles with peracetic acid and sodium hypochlorite was done by SEM. The samples were coated by Au/Pd sputtering to make the analyzed surfaces conductive. Then, a HITACHI S-2400 (Hitachi Instruments Inc., Tokyo, Japan) scanning electron microscope operating at 18 kV was used to take images of the surfaces of the samples.

#### 2.5.3. Energy-Dispersive X-ray (EDX) Spectroscopy Analysis

A qualitative analysis of the surface elemental composition of the same sanitized samples that were used in the SEM analysis was done by EDX spectroscopy using a Quantax 200 (Bruker Corporation, Billerica, MA, USA) spectroscope with an XFlash 5010/30 detector and the microanalysis software ESPRIT 1.9.

#### 2.5.4. X-ray Photoelectron Spectroscopy (XPS) Analysis

A quantitative analysis of the surface chemistry of the uncoated SS and coated AP10 + AA6 plates before and after 5 sanitization cycles with peracetic acid and sodium hypochlorite was done by XPS. Non-sanitized plates that were coated only with the APTES base coating (AP10) were also analyzed by XPS to better understand the changes that the sanitization processes provoked in the surface chemistry of the coating AP10 + AA6. Samples were analyzed in triplicate. A Kratos AXIS Supra system (Kratos Analytical Ltd., Manchester, UK) equipped with a monochromatic Al-Kα X-ray source (120 W, 8 mA/15 kV) operating at a pressure of 1.33 × 10^−7^ Pa was used for the acquisition of the XPS spectra. The survey and high-resolution spectra were acquired using pass energy settings of 160 eV and 20 eV, respectively. The binding energy of each spectrum was corrected by placing its C1s peak at 285 eV. The correction of the binding energy and the quantification of the atomic percentages of the main elements constituting the surface chemistry of the analyzed samples were done by means of the software CasaXPS 2.3.19 (Casa Software, Ltd., Teighmouth, UK). The high-resolution C1s regions of the spectra were deconvoluted using the software PeakFit 4.12 (SPSS Inc., Chicago, IL, USA).

#### 2.5.5. Fourier Transform Infrared Spectroscopy with Attenuated Total Reflectance (FTIR-ATR)

Coated samples that had been subjected to 5 sanitization cycles with each of the two solutions, as well as non-sanitized samples, were also analyzed by FTIR-ATR to ascertain if the chemical characteristics of the coating AP10 + AA6 were affected by the use of an acidic (peracetic acid) or alkaline (sodium hypochlorite) solutions. FTIR-ATR spectra with 4 cm^−1^ resolution were acquired in 32 scans by a Spectrum Two FT-IR spectrometer (PerkinElmer Inc., Waltham, MA, USA). Baseline correction of the FTIR-ATR spectra was performed with the software Spectrum 10.4.3.339 (PerkinElmer Inc., Waltham, MA, USA).

#### 2.5.6. Water Contact Angle (WCA) Measurement

In order to study how the wettability of the uncoated and coated surfaces was affected by the application of successive sanitization cycles, their static WCA before and after each cycle of sanitization was measured by the sessile drop method. Photographs of three distilled water drops on the surface of each SS plate were taken, and their WCA was measured using the free software ImageJ [21] and the low-bond axisymmetric drop shape analysis plugin [22]. The WCA of each sample was obtained by calculating the average of its three respective measurements.

## 3. Results and Discussion

### 3.1. Biofilm Measurements

**Figure 1:**
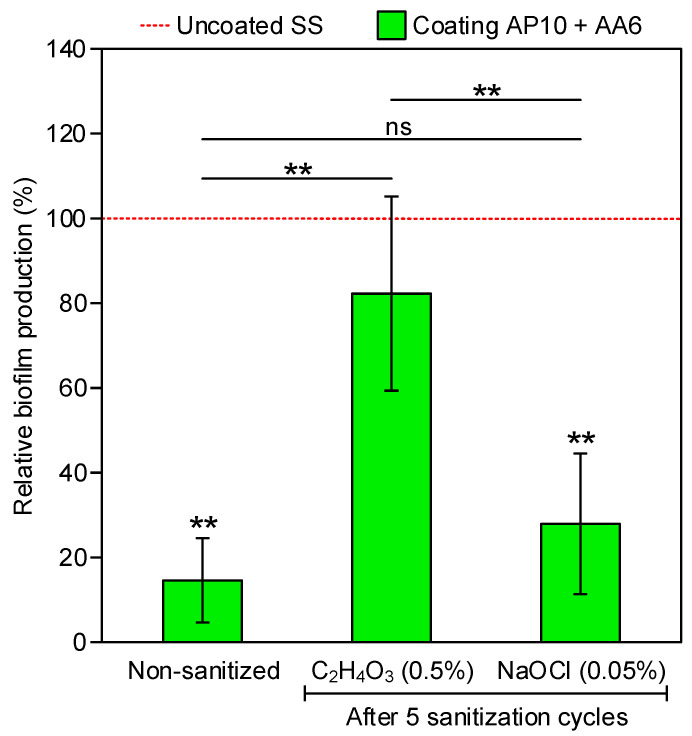
Relative biofilm production of the three-strain *L. monocytogenes* cocktail on uncoated SS and on the coating under the three different sanitization scenarios. The 100% relative biofilm production in each sanitization scenario corresponds to the biofilm formation observed on the uncoated SS plates. Statistically significant relationships (*p* < 0.05) are indicated with asterisks (**, *p* < 0.01).

Biofilm formation by a three-strain cocktail of *L. monocytogenes* on the uncoated and coated plates was quantified by CV staining. This quantification was made for uncoated SS and coated plates in three sanitization scenarios: (1) non-sanitized and after five sanitization cycles with (2) peracetic acid and (3) sodium hypochlorite. The coating exhibited anti-biofilm effectiveness before (i.e., non-sanitized) and after sanitization with both disinfectant solutions, presenting <100% relative biofilm production when compared with biofilms formed on the uncoated SS (Figure 1). However, this reduction in biofilm formation was statistically significant only for the non-sanitized coating and for the coating after sanitization with sodium hypochlorite. Relative biofilm productions of 14.6 ± 10% were obtained with the non-sanitized coating in comparison to the non-sanitized, uncoated SS. These relative biofilm production values were similar to those reported in our previous work [17] for a single-strain *L. monocytogenes* biofilm under the same incubation conditions, which confirms that this coating maintains its anti-biofilm effectiveness in a more realistic scenario using a multi-strain biofilm. On the other hand, after five sanitization cycles with peracetic acid, statistically significant greater relative biofilm production values than those of the non-sanitized coating were obtained, showing that the anti-biofilm effectiveness of the coating was diminished to some extent by this sanitization process. Nevertheless, the differences between the relative biofilm production on the non-sanitized coating and the coating after five sanitization cycles with sodium hypochlorite were not statistically significant, indicating that the anti-biofilm activity was not lost after this cleaning process.

### 3.2. AFM and SEM-EDX Analyses

**Figure 2:**
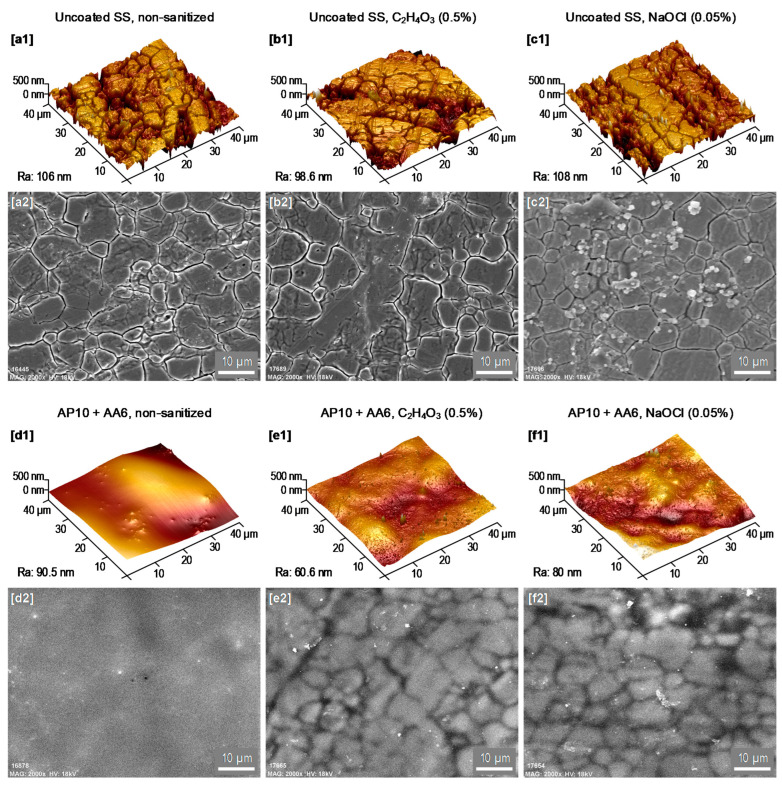
(**1**) AFM images (40 × 40 µm) and (**2**) SEM images (×2000) of (**a**–**c**) the uncoated SS and (**d**–**f**) the coating in three sanitization scenarios: non-sanitized and after five sanitization cycles with peracetic acid (0.5%) and sodium hypochlorite (0.05%).

**Figure 3:**
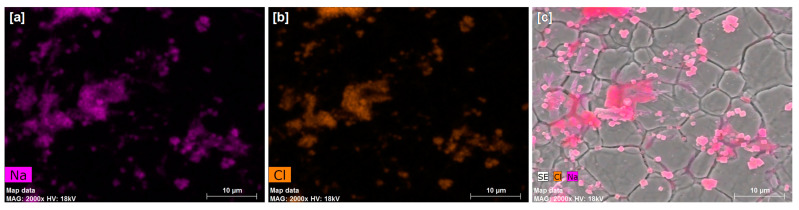
EDX and SEM images (×2000) of the uncoated SS after five sanitization cycles with sodium hypochlorite (0.05%): EDX maps of (**a**) sodium and (**b**) chlorine, and (**c**) EDX maps overlapped with the SEM image of the same analyzed area.

As shown in Figure 2, AFM and SEM images were taken at the surface of the uncoated SS and coated plates before sanitization (i.e., non-sanitized) and after five sanitization cycles with peracetic acid and sodium hypochlorite, whereas the uncoated SS (Figure 2a–c) showed the characteristic grooves of SS surfaces, the coated samples (Figure 2d–f) showed smoother surfaces where the presence of grooves was less evident in any of the three scenarios. These observations indicate that the coating AP10 + AA6 resisted, at least in part, the five sanitization cycles with both disinfectant solutions. It is also worth noting that the sanitization processes caused some changes in the coating AP10 + AA6 and the uncoated SS: (1) grooves of the SS substrate underneath the coating AP10 + AA6 became more evident in the SEM analysis after sanitization (Figure 2e2,f2) than before sanitization (Figure 2d2), suggesting a decrease in the coating thickness due to a partial removal of the coating by the sanitization processes; and (2) SEM images of the uncoated SS after sanitization with sodium hypochlorite (Figure 2c2) showed that its surface was populated with grainy structures which, as the EDX analysis of this surface (Figure 3) revealed, were composed of sodium and chlorine. These observations indicate that those grainy structures are sodium chloride (NaCl) crystals that originated from the sodium hypochlorite solution [23]. As concluded in our previous work [17], the morphology of the coating AP10 + AA6 contributed to its anti-biofilm effectiveness by reducing the occurrence of grooves on the SS substrate where bacteria would be prone to accumulate. According to the results of the morphological characterization tests of the current study, the sanitizing of the coating AP10 + AA6 with any of the two solutions resulted in smoother surfaces than those of the uncoated SS, with a reduced occurrence of grooves. Therefore, it can be considered that after the sanitization processes, the surface morphology of the coating still played a role in reducing biofilm formation through bacterial attachment prevention.

### 3.3. XPS Analysis

A quantitative analysis of the surface chemistry of the coated (AP10 + AA6) and uncoated SS plates before and after five sanitization cycles with each of the two sanitizing solutions was done by XPS. To better understand the possible deterioration of the coating by the sanitization processes, the surface chemistry of the non-sanitized base coating of APTES (AP10) was also analyzed. Atomic percentages of the elements detected on each studied surface are shown in Table 1.

Iron and chromium, which are characteristic elements of the SS AISI 316 substrate, were not detected on the surface of the coating AP10 + AA6 neither before nor after sanitization with any of the two solutions. This fact demonstrates that the coating completely covered the SS plates both before and after the sanitization processes. In addition, small percentages of nitrogen were measured on the surfaces of the uncoated SS plates because this element was present in the composition of the austenitic SS AISI 316 that was used to manufacture the plates of the current work. Compared to the uncoated SS, higher percentages of nitrogen were measured on the coated plates, which are explained by the incorporation of nitrogen coming from three main sources during the plasma-polymerization process: (1) the N_2_ plasma jet, (2) the APTES precursor of the base coating, and (3) the surrounding atmospheric air. After five sanitization cycles with any of the two solutions, greater atomic percentages of silicon and nitrogen than those before sanitization were measured on the coating. The increase in the atomic percentage of silicon (Si2p), which is a characteristic element of the base coating AP10, was especially remarkable. Whereas it was barely detected on the non-sanitized coating AP10 + AA6 (0.15%), its presence at the surface became more prominent after sanitization with the solutions of peracetic acid (4.51%) and sodium hypochlorite (5.86%). Nevertheless, neither silicon nor nitrogen reached the same atomic percentages on the coating AP10 + AA6 after sanitation as on the non-sanitized base coating AP10. Therefore, these results suggest that although the AcAc functional coating was deteriorated to some extent by the sanitization cycles, part of it still remained on the surface.

On the other hand, the presence of sodium and chlorine on both the coated and uncoated SS after sanitization with sodium hypochlorite indicated that this solution left some residues on the studied surfaces. Whereas the SEM-EDX analysis revealed that these residues were in the form of NaCl crystals on the uncoated SS (Figure 3), there were no remarkable surface features that could be attributed to sodium hypochlorite residues on the coated samples. It is known that metals such as Ni, Co, Cu, and Fe can catalyze the decomposition of sodium hypochlorite into NaCl [24]. Therefore, the formation of NaCl crystals only on the uncoated SS was likely due to the direct contact between the sodium hypochlorite solution and the SS surface, which would have facilitated the decomposition of the sodium hypochlorite.

As previously observed, oxygen-containing polar groups such as C-O, C=O, and O-C=O may play an important role in preventing the attachment of bacteria and proteins to a surface by inducing a hydrophilic character that promotes the formation of a water barrier between the surface and the microorganisms [17]. More specifically, the O-C=O group, which is associated with the carboxyl moiety (COOH) of the AcAc molecule, is especially effective in this regard. This is because carboxyl groups have higher polarity and are more hydrophilic than other oxygen-containing functional groups [25]. Therefore, the deconvolution of the C1s regions of the XPS spectra of the studied surfaces in this work was done to quantify the relative abundance of oxygen-containing polar groups (Table 2). The following components were found in the deconvolution: C-C and C-H bonds at ≈285 eV, C-O bonds at ≈286.4 eV, C=O bonds at ≈288.1 eV, and O-C=O bonds at ≈289.2 eV.

Regarding the uncoated SS surface, no significant differences were observed between its C1s region before and after five sanitization cycles with none of the disinfectants used. In addition, its relative percentages of polar oxygen-containing groups (24.14–29.81%) were considerably lower than those of the coated samples (43.18–54.64%). For the coating AP10 + AA6, a decrease in the relative percentages of polar oxygen-containing groups from that of the non-sanitized coating (54.64%) to values that were close to that of the base coating AP10 (46.41%) was measured after sanitization with both disinfectants. This was due mainly to a decrease in the relative percentage of the O-C=O group. Whereas this group accounted for 25.95% of the C1s XPS region of the non-sanitized coating AP10 + AA6, it was not detected on the surface of the base coating AP10. Therefore, the O-C=O group was characteristic of the functional coating of AcAc that was deposited over the base coating of APTES. In addition, it is worth noting that even after the sanitization processes, the O-C=O group was still found on the coating AP10 + AA6. These results suggested that a partial deterioration of the functional coating was caused by the sanitization processes, which is in accordance with our previous observations based on the atomic percentages of silicon and nitrogen.

The O/C ratio and the oxygenated/non-oxygenated carbon ratio were also calculated (Table 3). The former was obtained from the data in Table 1, dividing the values in the O1s column by those in the C1s column. The latter was obtained from the data in Table 2, dividing the values in the total polar groups column by those in the C-C and C-H column.

The higher the O/C ratio, the more inorganic a surface is. This is why the O/C ratios of the uncoated SS and the silicon oxide-based coating AP10 were generally greater than those of the coating AP10 + AA6. By comparing the values of the O/C ratio of the coating AP10 + AA6 in the three sanitization scenarios (i.e., the same coating with similar chemical characteristics in three different situations), one can infer that the greater the O/C ratio was, the more oxidized its surface was, which would be beneficial for the polarity, hydrophilicity, and anti-biofilm effectiveness of the coating. Although the differences between these O/C ratios were small because they were in the range of 0.39–0.50, they somehow agree with the biofilm production results (Figure 1), where the highest relative biofilm production (i.e., the lowest anti-biofilm effectiveness) was obtained after sanitizing coating AP10 + AA6 with peracetic acid, which was the scenario with the lowest O/C ratio. The relationship between the presence of polar oxygenated carbon groups and the anti-biofilm effectiveness of the coating AP10 + AA6 is clearer when the oxygenated/non-oxygenated carbon ratio is studied. As one can see in Table 3, this ratio was considerably greater in the coating AP10 + AA6 than in the uncoated SS in any of the three sanitization scenarios. Furthermore, by comparing the results of the coating in the three scenarios, one can see that the greater its oxygenated/non-oxygenated carbon ratio was (i.e., the more prominent the presence of polar groups), the lower its relative biofilm production was (i.e., the greater its anti-biofilm effectiveness).

### 3.4. FTIR-ATR Analysis

**Figure 4:**
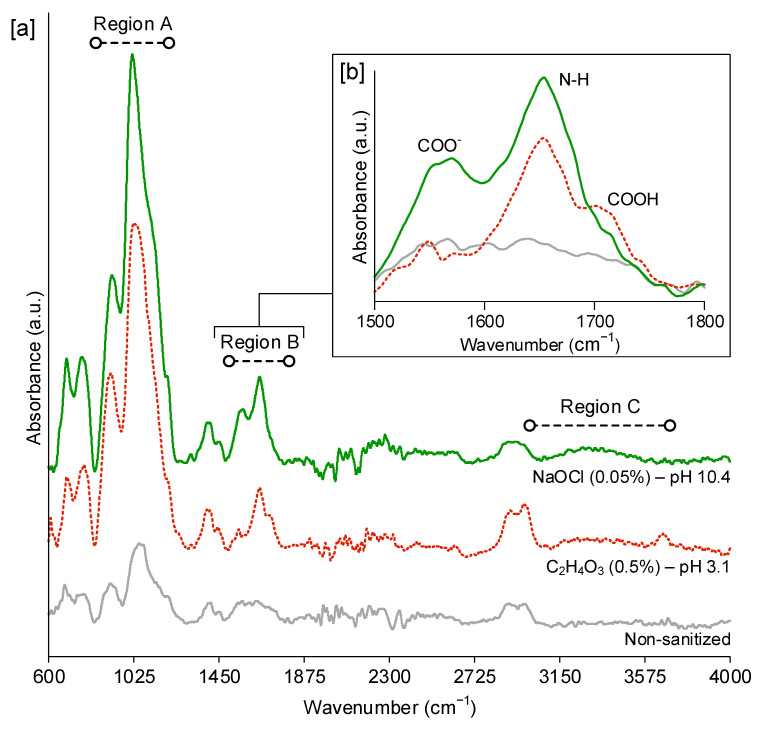
(**a**) FTIR-ATR spectra of the coating AP10 + AA6 non-sanitized (gray line) and after five sanitization cycles with sodium hypochlorite (0.05%) (green line) and peracetic acid (0.5%) (red dotted line); (**b**) close view of the region related with the presence of COOH groups and COO^−^ anions.

Carboxylic acid groups (COOH) are pH-sensitive, so carboxylic acid-based coatings, such as the functional AcAc coating of the current work, have different behaviors upon exposure to acidic (pH < 7) or alkaline (pH > 7) environments. Whereas COOH groups are uncharged at low pH conditions, they are deprotonated at high pH conditions and form carboxylate anions (COO^-^), which makes the polymeric coatings become more polar, more hydrophilic (i.e., showing lower WCA), and negatively charged [26,27]. Since most bacterial surfaces are negatively charged, the negative charge of the coating would promote an electrostatic repulsion that would reduce bacterial attachment [28,29]. The peracetic acid and sodium hypochlorite solutions presented very different pH values (pH 3.1 and pH 10.4, respectively), so the coating AP10 + AA6 was analyzed by FTIR-ATR spectroscopy to distinguish between the presence of COOH groups and COO^−^ anions. Figure 4a shows the full FTIR-ATR spectra of the coating AP10 + AA6 non-sanitized and after five sanitization cycles with each solution, and Figure 4b shows a close view of the region of 1500–1800 cm^−1^, where COOH groups and COO^−^ anions can be identified [30,31]. Three main regions were observed in the full FTIR-ATR spectra (Figure 4a). Region A (835–1300 cm^−1^) showed peaks at ≈915 cm^−1^ and ≈1040 cm^−1^, which were caused by the presence of Si-N and Si-O-Si groups. Region B (1500–1800 cm^−1^) showed a peak at ≈1650 cm^−1^ that was caused by the presence of amides and imines. Region C (3000–3700 cm^−1^) showed a broad band that was due to the NH of amine groups. These peaks and band are typical of APTES coatings [32], such as the base coating of the current work. The fact that they appeared in the spectra of Figure 4a indicates that the FTIR-ATR analysis, which has ≈1000 nm of analysis depth, reached the base coating. Furthermore, it is worth noting that those peaks and bands were more prominent in the sanitized samples than in the non-sanitized ones. This fact could be explained by the deterioration of the AcAc functional coating that was caused by the sanitization processes, which would have allowed the FTIR-ATR analysis to penetrate more in the APTES base coating of the sanitized samples. By taking a close view of Region B (Figure 4b), the COO^−^ anions and COOH groups could be studied. The peak at ≈1560 cm^−1^ was due to the presence of COO^−^ anions, and the shoulder that appeared at ≈1710 cm^−1^ in the FTIR-ATR spectra of the coating after sanitization with peracetic acid was due to the presence of COOH groups. The absence of the COOH shoulder in the spectra of the coating after sanitization with sodium hypochlorite and the presence of a more intense COO^−^ peak than that found after sanitization with peracetic acid confirmed that the degree of deprotonation of the functional coating was affected by the pH of the sanitizing solutions. Therefore, a higher degree of deprotonation of the carboxylic acid groups of the coating AP10 + AA6 was observed after sanitization with sodium hypochlorite than with peracetic acid.

### 3.5. WCA Measurements

**Figure 5:**
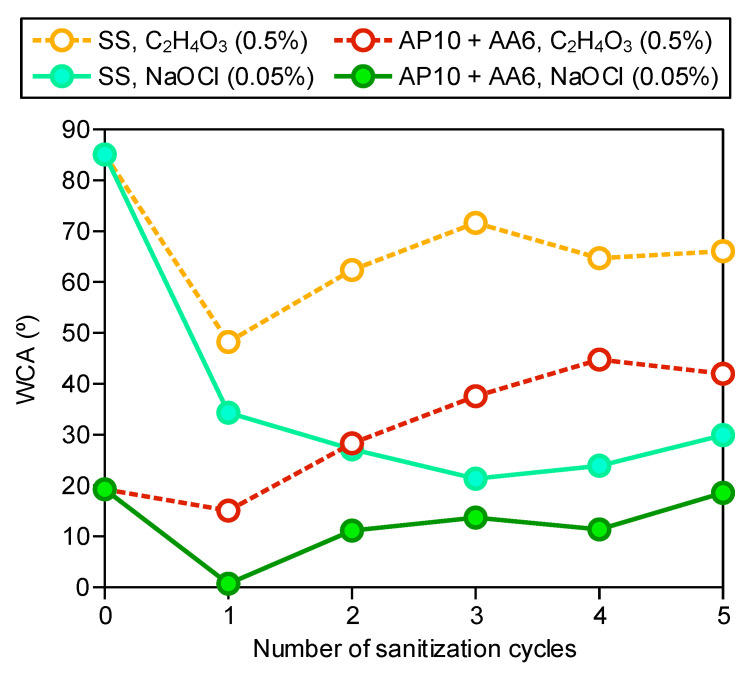
Evolution of the WCA of the uncoated SS and the coating AP10 + AA6 subjected to five sanitization cycles with peracetic acid (0.5%) and sodium hypochlorite (0.05%). The WCA values at zero sanitization cycles correspond to the non-sanitized surfaces.

According to our previous findings, the strong hydrophilicity of the coating AP10 + AA6 was one of the key factors contributing to its anti-biofilm effectiveness [17]. In the current work, the evolution of the WCA of the uncoated SS and the coating AP10 + AA6 after five sanitization cycles with peracetic acid and sodium hypochlorite was studied (Figure 5) to determine the changes in wettability that could induce variations in the production of biofilm on the surfaces.

For the uncoated SS, sanitization with any of the two disinfectant solutions reduced the WCA to some extent, thus making the surface more hydrophilic. Whereas a moderate reduction of the WCA of the uncoated SS from the initial 85° to ≈65° was measured after sanitization with peracetic acid, a considerable reduction to ≈30° was measured after sanitization with sodium hypochlorite. As shown by the SEM-EDX analysis (Figure 3), NaCl crystals were formed on the surface of the uncoated SS after sanitization with sodium hypochlorite. It is known that NaCl is hydrophilic, and its use for improving the hydrophilicity of other materials has been previously reported [33]. Therefore, the considerable decrease in the WCA of the uncoated SS that was sanitized with sodium hypochlorite can be explained by the presence of NaCl residues from this sanitizing solution after drying of the SS surface.

For the coating AP10 + AA6, whereas an increase in the WCA to ≈40° was measured after sanitization with peracetic acid, a strong hydrophilicity with WCA < 20° was maintained after sanitization with sodium hypochlorite. This can be explained by the different pH values of the sanitizing solutions. As observed in the FTIR-ATR analysis, the sodium hypochlorite solution (pH 10.4) caused a deprotonation of the carboxylic acid groups that resulted in a higher presence of COO^−^ anions than after sanitization with the peracetic acid solution (pH 3.1). This would favor the preservation of a stronger polarity and hydrophilicity after sanitization with sodium hypochlorite than after sanitization with peracetic acid. Therefore, from the results of the FTIR-ATR analysis and WCA measurements, it is concluded that the remarkable anti-biofilm effectiveness of the coating after five sanitization cycles with sodium hypochlorite, which resulted in a 27.9 ± 16.6% relative biofilm production (Figure 1), was due to the deprotonation of the remaining carboxylic acid groups. This deprotonation enabled the retention of a strong hydrophilicity (WCA < 20° in Figure 5) in spite of the partial deterioration of the functional coating, and would have provided the surface of the coating with an electronegative charge that facilitated the electrostatic repulsion of bacteria. On the other hand, the relatively low degree of deprotonation of the carboxylic acid groups after sanitization with peracetic acid seems to be less capable of compensating the deterioration of the functional coating. Thus, an 82.3 ± 22.9% relative biofilm production was obtained in this case, which implied a moderate anti-biofilm effectiveness.

Finally, it is also remarkable that after the first sanitization cycle of the coating AP10 + AA6 using the peracetic acid solution, a very similar WCA to that of the non-sanitized coating was observed, while when the sodium hypochlorite solution was used, the WCA considerably decreased to almost 0°. This decrease was likely due to the alkaline sodium hypochlorite solution causing further hydrophilization of the functional coating through the deprotonation of the COOH groups. From the second cycle onwards, the WCA of the coating showed an increasing trend when both sanitizers were used; which was probably due to the partial deterioration of the functional coating and the decrease in the oxygen-containing polar groups as observed in the XPS analysis. These results suggest that the first cycle did not provoke a noticeable deterioration of the functional coating and that it started to be partially degraded with the second cycle, although a more thorough chemical characterization comprising the state of the coating after different sanitization cycles would be necessary to confirm this assumption.

## 4. Conclusions

In this study, the anti-biofilm effectiveness of the plasma-polymerized coating AP10 + AA6 against biofilm formation by a multi-strain cocktail of *L. monocytogenes* has been demonstrated. In the non-sanitized coated plates, the relative biofilm production was 14.6 ± 10% in comparison to biofilm formation on uncoated plates. Furthermore, the anti-biofilm effectiveness of the coating AP10 + AA6 was retained to some extent after five sanitization cycles with peracetic acid and sodium hypochlorite solutions.

Whereas a moderate anti-biofilm formation activity was still obtained after sanitizing the coating with peracetic acid, a considerable reduction of biofilm production was still achieved after sanitization with sodium hypochlorite, which resulted in a relative biofilm production of 27.9 ± 16.6% in comparison to the uncoated SS. The results of the chemical characterizations suggest that the high pH of the sodium hypochlorite solution caused a higher degree of deprotonation of the carboxylic acid groups of the AcAc functional coating than the low pH of the peracetic acid solution. Thus, in spite of the partial deterioration of the hydrophilic functional coating of AcAc, a strong hydrophilicity (WCA < 20°) was retained after sanitization with sodium hypochlorite. Therefore, one of the main factors that prevented the initial bacterial attachment to the surface was preserved. Furthermore, the aforementioned deprotonation would have provided the surface of the coating with an electronegative charge, which may have contributed to prevent bacterial attachment by promoting the electrostatic repulsion of bacteria.

According to these observations, it can be concluded that the coating AP10 + AA6 is compatible with alkaline sanitizers, such as sodium hypochlorite. Therefore, the findings of the present study are a promising step forward to enable the industrial implementation of this coating.

Considering that the coating AP10 + AA6 is meant to be used in the food industry, including food-contact applications, its toxicity assessment is an important factor that still needs to be accomplished. Therefore, in order to ascertain the safety of this coating, future work will focus on identifying and evaluating any possible toxic effect. In addition, future works assessing the effectiveness of the coating using a multi-species biofilm should be carried out. Future perspectives for working with this coating also include its possible applicability in the dairy industry, where anti-biofilm surfaces are also a topic of interest. In this regard, the authors have planned experiments that involve coating cheese-making molds and plates for heat exchangers that will be tested in a dairy production pilot plant.

## Figures and Tables

**Table 1:** Elemental composition of the studied surfaces. Hyphens (-) indicate undetected elements.

Sample	Sanitizing Solution	Elemental Composition (%)							
		C1s	N1s	O1s	Si2p	Fe2p	Cr2p	Na1s	Cl2p
SS AISI 316	None	53.99	1.33	33.56	-	9.30	1.83	-	-
	C_2_H_4_O_3_ (0.5%)	47.37	2.13	30.32	-	15.68	3.59	-	-
	NaOCl (0.05%)	59.13	0.73	26.69	0.71	6.76	0.97	4.19	0.82
AP10 + AA6	None	66.70	2.52	30.63	0.15	-	-	-	-
	C_2_H_4_O_3_ (0.5%)	66.75	2.97	25.77	4.51	-	-	-	-
	NaOCl (0.05%)	58.33	3.49	28.91	5.86	-	-	2.07	1.34
AP10	None	48.55	5.10	35.21	11.13	-	-	-	-

**Table 2:** Relative percentages of surface chemical groups in the C1s XPS region of the studied samples.

Sample	Sanitizing Solution	Contribution in the C1s Region (at %)				
		C-C, C-H	C-O	C=O	O-C=O	Total Polar Groups (Sum of C-O, C=O, and O-C=O)
SS AISI 316	None	70.19	16.58	7.46	5.76	29.81
	C_2_H_4_O_3_ (0.5%)	72.30	17.99	4.69	5.02	27.70
	NaOCl (0.05%)	75.86	14.07	4.49	5.57	24.14
AP10 + AA6	None	45.36	28.33	0.36	25.95	54.64
	C_2_H_4_O_3_ (0.5%)	56.82	28.26	2.75	12.71	43.18
	NaOCl (0.05%)	52.81	25.21	15.36	6.62	47.19
AP10	None	53.59	36.36	10.05	-	46.41

**Table 3:** O/C ratio and oxygenated/non-oxygenated carbon ratio of the studied surfaces.

Sample	Sanitizing Solution	O/C Ratio	Oxygenated/Non-Oxygenated Carbon Ratio
SS AISI 316	None	0.62	0.42
	C_2_H_4_O_3_ (0.5%)	0.64	0.38
	NaOCl (0.05%)	0.45	0.32
AP10 + AA6	None	0.46	1.20
	C_2_H_4_O_3_ (0.5%)	0.39	0.76
	NaOCl (0.05%)	0.50	0.89
AP10	None	0.73	0.87

## Data Availability

No new data were created or analyzed in this study. Data sharing is not applicable to this article.
